# Treatment of Basal Cell Carcinoma Using a One-Stop-Shop With Reflectance Confocal Microscopy: Study Design and Protocol of a Randomized Controlled Multicenter Trial

**DOI:** 10.2196/resprot.4303

**Published:** 2015-09-10

**Authors:** Daniel J Kadouch, Albert Wolkerstorfer, Yannick Elshot, Biljana Zupan-Kajcovski, Marianne B Crijns, Markus V Starink, Marcel W Bekkenk, Allard C van der Wal, Phyllis I Spuls, Menno A de Rie

**Affiliations:** ^1^Academic Medical CenterDepartment of DermatologyUniversity of AmsterdamAmsterdamNetherlands; ^2^Netherlands Cancer InstituteDepartment of DermatologyAmsterdamNetherlands; ^3^Academic Medical CenterDepartment of PathologyUniversity of AmsterdamAmsterdamNetherlands; ^4^VU Medical CenterDepartment of DermatologyVU UniversityAmsterdamNetherlands

**Keywords:** carcinoma, basal cell, microscopy, confocal, diagnostic services, sensitivity and specificity, surgical procedures, operative

## Abstract

**Background:**

Basal cell carcinoma (BCC) is the most common cancer diagnosed in white populations worldwide. The rising incidence of BCC is becoming a major worldwide public health problem. Therefore, there is a need for more efficient management.

**Objective:**

The aim of this research is to assess the efficacy and safety of a one-stop-shop (OSS) concept, using real-time *in vivo* reflectance confocal microscopy (RCM) (Vivascope 1500; Lucid Technologies, Henrietta, NY, USA) as a diagnostic tool, prior to surgical management of new primary BCCs.

**Methods:**

This is a prospective non-inferiority multi-center RCT designed to compare the “OSS concept using RCM” to current standards of care in diagnosing and treating clinically suspected BCC. Patients ≥ 18 years attending our outpatient clinic at the Department of Dermatology, Academic Medical Center, University of Amsterdam, and the Department of Dermatology, the Netherlands Cancer Institute-Antoni van Leeuwenhoek Hospital (Amsterdam, The Netherlands) with a clinically suspected new primary BCC lesion will be considered for enrollment using predefined inclusion and exclusion criteria, and will be randomly allocated to the experimental or control group. The main outcome parameter is the assessment of incomplete surgical excision margins on the final pathology report of confirmed BCC lesions (either by punch biopsy or RCM imaging). Other outcome measures include diagnostic accuracy (sensitivity and specificity) of RCM for diagnosing BCC and dividing between subtypes, and throughput time. Patient satisfaction data will be collected postoperatively after 3 months during routine follow-up.

**Results:**

This research is investigator-initiated and received ethics approval. Patient recruitment started in February 2015, and we expect all study-related activities to be completed by fall 2015.

**Conclusions:**

This RCT is the first to examine an OSS concept using RCM for diagnosing and treating clinically suspected BCC lesions. Results of this research are expected to have applications in evidence-based practice for the increasing number of patients suffering from BCC and possibly lead to a more efficient disease management strategy.

**Trial Registration:**

ClinicalTrials.gov: NCT02285790; https://clinicaltrial.gov/ct2/show/NCT02285790 (Archived by WebCite at http://www.webcitation.org/6b2LfDKWu).

## Introduction

### Basal Cell Carcinoma

Basal cell carcinoma (BCC) is the most common cancer diagnosed in white populations worldwide. The rising incidence of BCC is becoming a major worldwide public health concern [1,2]. Between 1973 and 2009, the European standardized rate quadrupled from 40 to 165 per 100,000 person-years for men and from 34 to 157 for women, most likely because of more intensive UV exposure [3]. This is supported by previous published epidemiological literature indicating that ultraviolet radiation is an important risk factor for BCC, with a significant increase among outdoor workers [4,5]. Despite the low mortality from BCC, multiple and recurring tumors confer a high morbidity and considerable burden for health care providers and health budgets. Although BCC does not seem to have a strong effect on patients’ quality of life, patients suffering from BCC are definitely interested in efficacy, low recurrence rates, and cosmetic outcomes of their treatment [6]. Meanwhile, resources available at hospitals have not increased proportionally, and therefore, optimizing the effectiveness of present treatment modalities in daily dermatologic practice is necessary [7].

Clinically, BCC are characterized by small, translucent, or pearly papules, with raised teleangiectatic edges [8]. Most BCC occur in sun-exposed skin of the head and neck areas [9,10]. Sensitivity and positive predictive value of the clinical diagnosis of BCC by dermatologists have been reported to be 95.4% and 85.9%, respectively [11]. However, dividing between BCC subtypes is not always possible upon clinical assessment. To date, histological analysis of punch biopsy remains the gold standard to confirm the clinical diagnosis of BCCs and divide between the following subtypes: superficial (sBCC), nodular (nBCC), micro nodular (mnBCC) and infiltrating (iBCC). Of those, nBCC and sBCC have a less aggressive growth pattern in comparison to mnBCC and iBCC. Additionally, mixed type BCC (mtBCC) can be defined as a combination of subtypes and is frequently composed of aggressive subtypes [12]. Surgical excision remains the standard of treatment, with Mohs micrographic surgery typically utilized for high-risk lesions [13]. Based upon the histological growth pattern, BCC are surgically removed with a margin of either 3 mm (nBCC and sBCC) or 5 mm (mnBCC and iBCC) in accordance with current Dutch guidelines.

### Reflectance Confocal Microscopy

The use of real-time *in vivo* reflectance confocal microscopy (RCM) has proven successful to noninvasively diagnose BCC. Various studies have demonstrated that RCM is safe and accurate (sensitivity and specificity) to diagnose BCC [14-18]. Reported sensitivity and specificity for RCM in diagnosing BCC range from 83%-100% and 79%-97%, respectively [19-25]. Furthermore, Peppelman et al and Longo et al recently reported on RCM features that might divide between nodular, micronodular, superficial, and infiltrative subtypes of BCC [24,26,27].

### One-Stop-Shop

In 2012, van der Geer et al reported on the feasibility of a one-stop-shop (OSS) concept for the treatment of skin cancer patients [28]. One-stop-shop implies that on the day of the initial outpatient clinic consultation, diagnosis and treatment planning both take place. In their study, preoperative frozen section histology was used to confirm BCC diagnosis and subtype. The mean throughput time was 4 hours and 7 minutes, no complications were observed, and patient satisfaction was high [28]. Incorporating RCM as a noninvasive diagnostic tool in a BCC OSS concept for lesions suitable for conventional surgical excision might further reduce the time between clinical diagnosis and treatment, administrative workload, and costs.

### Aims and Objectives

The aim of our study is to assess the efficacy and safety of the OSS concept, using real-time *in vivo* RCM (Vivascope 1500; Lucid Technologies, Henrietta, NY, USA) as a diagnostic tool, prior to the surgical management of new primary BCC, of all subtypes, in the general population. We hypothesize that compared to current standards of care, the OSS concept using RCM will not result in a significant increase of incomplete surgical excision margins on the final pathology report of confirmed BCC lesions. It is further hypothesized that in this OSS concept, RCM will have acceptable diagnostic accuracy (sensitivity and specificity) for diagnosing BCC and dividing between subtypes, throughput time will not increase, and patient satisfaction will be higher for participating subjects.

## Methods

### Recruitment, Screening, and Enrollment

Patients will be recruited from the outpatient clinics of the Department of Dermatology, Academic Medical Center, University of Amsterdam (AMC), and the Department of Dermatology, the Netherlands Cancer Institute-Antoni van Leeuwenhoek Hospital (AVL), second-line and third-line reference centers. Consecutive patients with clinically suspected new primary BCC will be prospectively enrolled and randomly assigned to either the experimental (RCM-OSS) or control (standard of care) group during times the study associates will be available. Clinical assessment will be performed by an experienced, board-certified dermatologist. Clinical and dermoscopy pictures of the BCC lesion will be taken by a medical photographer. Patients with multiple clinically suspected new primary BCC lesions will be included for only the lesion most suitable for conventional surgical treatment according to the following order: (1) chest, (2) extremities, and (3) head and neck area.

The inclusion criteria are the following:

1. patient with clinically suspected new primary BCC as assessed by an experienced board certified dermatologist, (2) age ≥18, (3) patient is willing and able to give written informed consent, (4) BCC lesion is suitable for conventional surgical excision under local anesthetics, and (5) BCC lesion has been present for at least 1 month.
2. The exclusion criteria are the following: (1) BCC lesion in a high-risk location of the face (H-zone and ears), (2) contra-indication for conventional surgical excision (primary surgical closure seems not achievable), (3) recurrent BCC lesion (BCC that has been previously unsuccessfully treated), (4) macroscopic ulcerating BCC lesions (not feasible for RCM analysis due to technical reasons), (5) patient with basal cell nevus syndrome, (6) patient treated with hedgehog inhibitor medication, (7) patient with a history of hypersensitivity and/or allergy to local anesthesia, (8) patient unavailable in the following 6 weeks (for example due to holidays or sports), and (9) patient not able to understand the procedures involved.

The investigators will enrol subjects at both study locations (AMC and AVL). Included patients with clinically suspected new primary BCC lesions will be randomly allocated to the different diagnostic procedures. The investigators will obtain the patient’s consent. Each consecutive patient will be assigned a randomization number according to a computer-generated randomization list (ALEA) using random block sizes of 2, 4, 6, and 8 to ensure treatment concealment. Randomization will take place between the control and experimental group. This study will have an open label set-up. The patient and local investigator will not be blinded.

The randomization will be blinded. The pathologists analyzing the final excision specimen will be blinded to the patient’s history and to the results of RCM imaging. Whenever the histology of the punch biopsy is not required in the diagnostic process of the final excision specimen, the pathologist will also be blinded for those results. After initial RCM diagnosis by the study associates (DK and YE), two independent outcome assessors (M. Ulrich, Charite Berlin in Germany and C. Longo, Modena and Reggio Emilia in Italy) analyzing the RCM images will be blinded to the patient’s history and to the results of the final pathology report (reference standard).

We chose a cutoff of 95% as an acceptable radical BCC excision rate with standard of care based on our experience. Using the Miettinen and Nurminen confidence interval around the risk difference (24), with two groups of 38 patients, we will have 80% power to assess noninferiority of the OSS concept with RCM over usual care, considering an expected radical BCC excision rate of 95% in both arms, a noninferiority limit (delta) of 15%, and a one-sided alpha of 0.05. 

### Outcome Measures

Incomplete surgical excision on the final pathology report of a routinely processed tissue specimen of confirmed BCC lesions (either by punch biopsy or RCM imaging) is the main outcome parameter. Assessment will be performed by an experienced board-certified pathologist. The number of incomplete excisions will be compared between the experimental and control group. Other assessments of included subjects with confirmed BCC lesions (either by punch biopsy or RCM imaging) will include the following:

1. Diagnostic accuracy (sensitivity and specificity) of the RCM for BCC diagnosing and subtyping will be separately analyzed by comparing RCM diagnosis and subtype with final pathology reports of the experimental group. This will be performed by using unidentifiable saved RCM images of all included lesions of the experimental group to analyze inter and intraobservership variability in the interpretation of RCM imaging. The study associates (DK and YE) and two independent outcome assessors (MU and CL) will be blinded to the patient’s history and to the results of the final pathology report (reference standard).
2. Throughput time will be assessed by the study associates and compared between the experimental and control group.
3. Patient satisfaction will be assessed postoperatively 12 weeks after excision by using a standardized web-based questionnaire for patient reported outcomes in the management of skin diseases. An adjusted version of this web-based questionnaire has previously been published to assess patient satisfaction among patients suffering from psoriasis [29]. The outcome of the questionnaire will be compared between the experimental and control group.
4. The frequency of and reasons for exclusions will be documented.
5. The frequency of interpretable, indeterminate, and intermediate tests will be documented.
6. Adverse events during the procedure will be documented.

### Study Procedures

BCCs will be divided into 5 main subtypes based on the histopathological growth pattern of the final excision specimen: superficial (sBCC), nodular (nBCC), micronodular (mnBCC), infiltrating (iBCC), and basosquamous (bBCC). In the case of mixed-type diagnosis, defined as two or more single growth patterns, the histology will be classified into single subgroups determined by the most aggressive component of the pathological feature according to the descending gradation from bBCC, iBCC, mnBCC, nBCC, to sBCC. The most aggressive component will determine the excision margin (5 mm versus 3 mm).

After obtaining written informed consent, the screening will be completed. Patients with clinically suspected new primary BCC lesions will be randomly allocated to the following regimes:

1. Experimental group (N=38): Clinically suspected new primary BCC lesions will be diagnosed and divided into subtypes using RCM imaging (Vivascope 1500; Lucid Technologies, Henrietta, NY, USA) according to a standardized protocol [24,26,27] (Table 1). After diagnosis, excision of BCC lesions with adequate margins will be performed on the same day at the Department of Dermatology according to the one-stop-shop concept. Clinically suspected primary BCCs that are not confirmed by RCM will also receive surgical treatment with a margin of 3 mm.
2. Control group (N=38): Clinically suspected new primary BCC lesions will be diagnosed and divided into subtypes according to current standards of care. A conventional 3 mm punch biopsy will be performed in the most elevated part of the lesion using local anesthetics (1% xylocaine/adrenaline). A biopsy specimen will be analyzed by a pathologist (within 2 weeks). After diagnosis, excision of the BCC lesions with adequate margins will be performed within the following 4 weeks according to current standards of care. Clinically suspected primary BCCs that are not confirmed by punch biopsy will also receive surgical treatment with a margin of 3 mm.
  The study design incorporated five parts. First, screening took place. Second, intake involved the following steps: written informed consent, intake, randomization, and photo documentation. Third, allocation to the experimental or control group consisted of (1) assessment of diagnosis and subtyping of clinically suspected new primary BCC, and (2) assessment of surgical margins. Fourth, surgical excision of the lesion took place: the excised surgical specimen was assessed by the pathologist and an assessment of throughput time was conducted. Finally, a routine 12-week postoperative control visit was conducted, involving an assessment of patient satisfaction using the web-based questionnaire (Multimedia Appendix 1).

**Table 1:** Expected RCM features of different BCC subtypes as previously reported in the literature.

Subtype	Epidermis	DEJ^a^	Upper dermis
sBCC	Epidermal streaming	cords connected to the epidermis that may occasionally display clefting and peripheral palisading of nuclei	thin blood vessels parallel to the en-face plane of RCM imaging
		OR	
		dark silhouettes embedded in stroma of thickened collagen	
		dilated blood vessels coursing parallel to en-face plane of imaging	
nBCC	Possible ulceration	increase in vascular diameter without cords connected to the epidermis	rounded to polycyclic basaloid bright tumor islands (*large in size*) with peripheral palisading of nuclei and surrounding dark clefting; stroma of thickened collagen
mnBCC	Possible ulceration	increase in vascular diameter without cords connected to the epidermis	rounded to polycyclic basaloid bright tumor islands (*smaller in size*) with peripheral palisading of nuclei and surrounding dark clefting; stroma of thickened collagen
iBCC		increase in vascular diameter without cords connected to the epidermis	the absence of small or big tumor islands
bBCC		no features previously reported	no features previously reported

^a^dermal epidermal junction

### Data Analysis

Data will be recorded on data entry forms and will be entered in a computer system for subsequent tabulation and statistical analysis. The data will be handled confidentially and anonymously. Furthermore, all information relevant to the treatment will be recorded in the electronic medical file.

All data will be collected and transferred to a Microsoft Excel database. The statistical analysis will be performed at the AMC using SPSS version 21.0. We will calculate the observed difference as the proportion of radical BCC excisions in the care-as-usual arm minus this proportion in the OSS with RCM arm, and calculate a one-sided 95% (or two-sided 90%) confidence interval for this difference using the Miettinen and Nurminen method [30]. The inferiority hypothesis will be rejected when the upper limit of this confidence interval does not exceed 15%. Side effects will be described per item.

## Results

This is an investigator-initiated unfunded prospective open-label noninferiority randomized controlled multicenter trial. Development of the project commenced in fall 2012, and the study protocol has been approved by the ethics committee at the coordinating center (AMC, METC 2014_244) and by the local Institutional Review Board at the participating center (AVL) in fall 2014. This trial has also been registered publically at ClinicalTrials.gov (identification number: NCT02285790). Patient recruitment started in February 2015, and the expected date of completion is fall 2015.

The study is being conducted according to the principles of the Declaration of Helsinki (Fortaleza, Brazil, October 2013) and in accordance with the Medical Research Involving Human Subjects Act (WMO) and other relevant guidelines, regulations, and acts.

## Discussion

BCC is the most prevalent skin cancer, and its prevalence is increasing [1]. Histological analysis of punch biopsy remains the gold standard to confirm a clinical diagnosis of BCC and dividing subtypes. However, due to the rising incidence of BCC, there is a need for more efficient, noninvasive methods of diagnosis. Incorporating RCM as a noninvasive diagnostic tool in a BCC OSS concept for lesions suitable for conventional surgical excision, in concordance with current Dutch guidelines, might reduce time between clinical diagnosis and treatment, administrative workload, and costs. Surgical treatment of BCC is generally performed under local anesthesia, which makes it suitable for an OSS approach.

Subjects participating in the study will be informed and will have to provide written informed consent prior to enrollment. Study participation will not result in additional follow-up visits other than clinically required 3 months postoperative.

Real-time *in vivo* RCM uses a confocal microscope to noninvasively image a thin surface of the skin at high resolution directly without the need for invasive biopsies. The diagnostic procedure itself is painless and no side effects have been reported. Outcome measures involve routinely processed surgical specimens after excision, patient satisfaction, calculation of throughput time, and analyzing diagnostic accuracy of the RCM procedure in subtyping BCC lesions. The overall burden of the study is minimal. A possible inconvenience for participating patients in the experimental group is that specific features for BCC subtyping are still being established. Therefore, a potential side effect for those patients may be less accurate subtyping of BCCs resulting in less adequate surgical margins. At the same time, RCM imaging may be of additional value in scanning the complete lesion, which potentially could prevent missing a more aggressive part of a tumor in contrast to a biopsy.

Thus, there is a potential benefit for the participating subject, namely noninvasive confirmation of clinically suspected BCC lesions followed by direct surgical treatment. Considering the relatively quick and simple procedure, noninvasiveness of the diagnostic method, and the one-stop-shop concept of diagnosing and treating BCC at the same consultation, the balance between burden, possible side effects, and prospect for improvement might be very favorable.

 This RCT is the first to examine an OSS concept using RCM for diagnosing and treating clinically suspected BCC lesions. Results of this research are expected to have applications in evidence-based practice for the increasing number of patients suffering from BCC, and possibly lead to a more efficient disease management strategy.

## Multimedia Appendix 1

Patient satisfaction questionnaire.
