# Supplementary material for: Treatment of Basal Cell Carcinoma Using a One-Stop-Shop With Reflectance Confocal Microscopy: Study Design and Protocol of a Randomized Controlled Multicenter Trial
Source: JMIR Res Protoc. 2015 Sep 10;4(3):e109. doi: 10.2196/resprot.4303 (PMC4704900; doi:10.2196/resprot.4303)
Supplement: Supplementary file 1 [file resprot_v4i3e109_app1.pdf]

Appendix 1: Questionnaire

Note: in the web-questionnaire, one item per page was shown. It was not possible to continue without answering a question. Respondents were able to review and change their answers through a Back button.

**Satisfaction with current treatment**

The following 5 questions concern your satisfaction with your current treatment.

1. How satisfied are you with your current treatment?

Please tick the number of your choice. 1=not at all satisfied, 5=very satisfied. Numbers 2, 3 and 4 are in between.

**Not at all satisfied**				**Very satisfied**
1	2	3	4	5

Characteristics of satisfaction

Your satisfaction with your treatment depends on multiple characteristics. For example, the effectiveness, safety, convenience, doctor-patient communication, information provision, and organization of treatment. The following questions concern those characteristics.

2. How satisfied are you with the effectiveness of your current treatment?

You may think of:
- successful treatment of the basal cell carcinoma lesion
- improvement of your quality of life, for example your mood, your vitality, how much time you are able to spend on working, hobbies or social contacts.

Please tick the number of your choice. 1=not at all satisfied, 5=very satisfied. Numbers 2, 3 and 4 are in between.

Not at all satisfied				Very satisfied
1	2	3	4	5

3. How satisfied are you with the safety of your current treatment?

You may think of the risk of side effects of the treatment, the risk to develop other diseases/complaints due to the treatment.

Please tick the number of your choice. 1=not at all satisfied, 5=very satisfied. Numbers 2, 3 and 4 are in between.

Not at all satisfied				Very satisfied
1	2	3	4	5

4. How satisfied are you with the convenience of your current treatment?

You may think of discomfort of the diagnostic procedure, waiting time spent in the hospital, waiting time between diagnosis and treatment of basal cell carcinoma lesion.

Please tick the number of your choice. 1=not at all satisfied, 5=very satisfied. Numbers 2, 3 and 4 are in between.

Not at all satisfied				Very satisfied
1	2	3	4	5

5. How satisfied are you with the information provision about your current treatment?

You may think about oral or written information about your treatment.

Please tick the number of your choice. 1=not at all satisfied, 5=very satisfied. Numbers 2, 3 and 4 are in between.

**Not at all satisfied**				**Very satisfied**
1	2	3	4	5

Treatment satisfaction in general

Characteristics of satisfaction

Your satisfaction with treatment depends on several characteristics. Some examples we mentioned before are effectiveness, safety, convenience, doctor-patient communication, information provision, organization of treatment. We are curious how important those characteristics are to you in choosing a treatment.

In answering the following question, please note that it concerns your opinion ***in general****, not only your current treatment.*

6. How important are those characteristics in general to you in choosing a treatment?

You have 10 points to divide. Please divide those over the characteristics. The more important a characteristic is in your opinion, the more points you give. If a characteristic is not important at all in your opinion, you give it zero points.

	**Number of points**
**Effectiveness**<br>You may think of:<br>- successful treatment of the basal cell carcinoma lesion<br>- improvement of your quality of life, for example your mood, your vitality, how	

much time you are able to spend on working, hobbies or social contacts.	
**Safety**<br>You may think of the risk of side effects of the treatment, the risk to develop other diseases/complaints due to the treatment.	
**Convenience**<br>You may think of discomfort of the diagnostic procedure and the time of uncertainty before diagnosis of basal cell carcinoma.	
**Information provision**<br>You may think about oral or written information about your treatment.	
**Doctor-patient communication**<br>You may think of the way the doctor chooses a particular treatment, in dialogue with you, the contact with your doctor during the consultations, the doctor’s attitude or how he/she treats you.	
**Organization of treatment**<br>You may think of the method of treatment (outpatient clinic, daycare center, in hospital or at home), the administration and how counter employees and other personnel (e.g. nurses) treat you during the treatment, possible waiting times, time between diagnosis and treatment.	
